# Guinea-Worm

**Published:** 1890-09-27

**Authors:** 


### GUINEA-WORM.

"The Guinea-Worm in Europe" is the rather sensational heading of a note recently furnished by a contemporary. It simply, however, appears that a medical practitioner in Brussels had two patients suffering from this parasite among the members of a troupe of negroes from Accra, on the Guinea Coast, who were performing in Brussels. Fortunately guinea-worm has not a very extensive habitat. It occurs throughout India, especially Western India. It is found on the shores of the Red Sea, particularly on the Arabian coast. It presents itself in Egypt and in some part of Equatorial Africa, and also on the Guinea coast and along the banks of the Niger River. When seen in European countries it is only in the persons of those who have contracted it in the East, or in Western Africa. The guinea-worm, *dracunculus*, or *filaria medinensis*, is a nematoid parasite, usually measuring from one to three feet in length, and having an inside sac, stretching along its whole extent, crowded with innumerable young, which Bastain asserted are produced without love, courtship, or matrimony, as the result of a non-sexual process. The guinea-worm has been the subject of much investigation and discussion in the East. Carter found that minute worms having great resemblance to the young of the guinea-worm existed in abundance in the mud tanks and wells near Bombay, and it was theorised that these worms entered the persons of those bathing in such waters, or going barefoot down the steps of tanks, as natives are in the habit of doing for water. Many arguments were brought forward in favour of this view, although it could not but be admitted that guinea-worms occurred in people who did not frequent the wells and tanks. The theory was finally disposed of by Sir William Moore, who drew attention to the fact that guinea-worm was prevalent in the semi-desert districts of Western Rajpootana, where water is hundreds of feet from the surface, where there are no steps to wells, and consequently where there is no mud for the development of the worm.

More recently the Russian surgeon, Fedschenki, advanced that escaped embryos of the guinea-worm perforate the skin of minute aquatic crustaceans, where they undergo further development. After a month they obtain their highest larval state of growth, within the crustacean, and along with their intermediate hosts are conveyed into the human stomach. But, again, Sir William Moore pointed out that these minute aquatic crustaceans, or cyclops, are not found in many localities where guinea-worm is well known. The fact is, that we are ignorant of the life cycle of the guinea-worm, and also of the mode in which it gains entrance into the system.

We may, however, be thankful that it is not a disease of this country, for if not successfully extracted, and this cannot always be accomplished, inflammations and suppurations occur, which cause much suffering, which have resulted in the disorganization and loss of limbs, and which have terminated fatally.

